# Starvation Ketoacidosis Induced by Ketogenic Diet and Consumption of Ketone Supplement

**DOI:** 10.7759/cureus.15778

**Published:** 2021-06-20

**Authors:** Manjot S Malhi, Frank Duerson, Joshua K Salabei, Peters Okonoboh

**Affiliations:** 1 Internal Medicine, University of Central Florida College of Medicine, Graduate Medical Education/North Florida Regional Medical Center, Internal Medicine Residency Program, Gainesville, USA; 2 Internal Medicine - Critical Care, University of Central Florida College of Medicine, Graduate Medical Education/North Florida Regional Medical Center, Internal Medicine Residency Program, Gainesville, USA

**Keywords:** keto diet, starvation ketoacidosis, starvation ketosis, chronic kidney disease (ckd), diabetes, metformin

## Abstract

The ever-changing landscape of dieting and its correlation with health outcomes have continued to evolve with time. New diets appear and disappear just as quickly as they gain notoriety. This is a rare case of a 67-year-old female with a history of type II diabetes who presented with generalized weakness, nausea, and vomiting, and was found to have severe anion gap metabolic acidosis. In an effort to lose weight, she was combining a ketogenic diet with prolonged fasting and exogenous ketone supplement use that she purchased online. The patient reported drinking an exogenous ketone ester supplement that contained 30 g of D-beta hydroxybutyrate (BHB) per serving, three times per day. This case is unique in that the patient was initially thought to be in diabetic ketoacidosis upon arrival, but after further investigation into her initial labs, medication, and social history, the underlying factor for hospitalization became evident; that is, a combination of a ketogenic diet with prolonged fasting and exogenous BHB-induced ketoacidosis in the setting of type II diabetes. Thus, this case highlights the importance of thorough history taking, the dangers of over-the-counter supplement consumption, and the risks consumers inherit with trend dieting.

## Introduction

Although traditionally used to treat epilepsy and other neurological conditions over the past decades, the ketogenic diet has gained popularity for its weight-reducing effects [1,2]. The basic foundation of this diet is to eliminate nearly all carbohydrate intake, reduce protein-sourced calories, and consume 70% or more of one’s daily caloric intake from fats. Thus, the ketogenic diet ultimately causes a state of insulin deficiency which allows for the production of ketones in the liver by a process called ketogenesis [3]. One of the main benefits of ketones is their ability to act as an alternative energy source to glucose. Blood ketone levels above 0.5 mM indicate a state of ketosis [4].

Under certain circumstances, a ketogenic diet coupled with a prolonged fasting state and ingestion of exogenous ketones can quickly induce a state of ketoacidosis. When this occurs, the patient’s serum bicarbonate levels decrease along with the pH leading to metabolic acidosis [5-7]. Although ketoacidosis is commonly associated with diabetes, there are other causes of ketoacidosis including, but not limited to, the use of sodium-glucose co-transporter 2 (SGLT2) inhibitors, alcohol, and starvation.

## Case presentation

We present the case of a 67-year-old female with a history significant for type II diabetes mellitus and obesity (body mass index of 43) who had been trying to lose weight with a combination of intermittent fasting and consumption of exogenous ketone supplements purchased online. Three months prior to hospitalization, the patient began a ketogenic diet that entailed intermittent fasting, whereby she would fast for upwards of 40 hours at a time with only drinking water and ingesting keto supplements; she reported drinking the ketone ester supplement three times a day on an empty stomach. The patient stated she had lost over 25 pounds since the beginning of her diet. After one of her fasting cycles, she ate half an avocado, some shrimp, and drank a ketone ester drink. Shortly after, the patient began feeling dizzy and started to vomit and ultimately presented to the hospital with severe fatigue, lower abdominal pain, nausea, and vomiting. She last took metformin (she was on a 750 mg twice daily home dose) on the morning of her presentation to the hospital and admitted to taking exogenous ketone supplements containing 30 g of beta-hydroxybutyrate (BHB) three times a day.

On evaluation, she was hemodynamically stable with baseline normal mental function but appeared dehydrated. Assessment of vitals showed the patient was tachycardic with a pulse rate of 120 beats per minute and borderline tachypnea with a respiratory rate of 20 breaths per minute. Her electrocardiogram did not show any arrhythmias or concerning changes in ST segments. On examination, she had dry mucous membranes and diffuse lower abdominal pain to palpation without guarding or rebound tenderness. Laboratory evaluation was notable for a white blood cell count of 30,100/mm^3^ (normal range: 4,500-11,000/mm^3^), creatinine 1.47 mg/dL (0.60-1.30 mg/dL), blood urea nitrogen 32 mg/dL (7-18 mg/dL), glucose 176 mg/dL (74-106 mg/dL), serum bicarbonate 8 mEq/L (21-32 mEq/L), anion gap 27.7 mEq/L (3-15 mEq/L), and a serum lactic acid of 2.9 mmol/L (0.4-2.0 mmol/L). The patient also had a moderately positive serum qualitative acetone level, plasma osmolality 312 mOsm/kg (275-301 mOsm/Kg), sodium 136 mEq/L (136-145 moml/L), and HbA1C of 8.4% (<5.7%). A urinalysis showed elevated urine ketones and was negative for glucose. Her pH from a venous blood gas analysis on admission was 6.81.

Differential diagnoses of diabetic ketoacidosis, alcoholic ketoacidosis, lactic acidosis, and salicylate toxicity were investigated. Serum and urine drug screens were negative for ethanol, acetaminophen, and salicylates. The patient was started on intravenous fluids with dextrose 5%-0.9% normal saline, sliding scale insulin, and 50 meq sodium bicarbonate 8.4%. After two days in the intensive care unit, the patient clinically felt much better. Her anion gap closed and her pH on a venous blood gas analysis was 7.38. The patient was transferred to the floors and was discharged the following day.

## Discussion

Ketosis is a metabolic state characterized by elevated levels of ketone bodies in the blood or urine. Ketone bodies, that is, acetoacetate, 3-BHB, and acetone are the predominant ones produced during ketogenesis. Ketogenesis occurs in the liver initiated in a hypoinsulinemic state. Low insulin levels due to either absolute or relative hypoglycemia as in a fasted state activate hormone-sensitive lipase that causes lipolysis releasing fatty acids that are transported to the liver ultimately producing ketone bodies [5,8].

The goal of ketosis in conditions such as fasting and ketogenic diets where the carbohydrate intake is severely limited is to provide an alternative source of energy other than glucose. Studies have shown that the ketogenic diet, which is designed as a low-carbohydrate and high-fat diet can induce ketosis and help in weight loss [3]. It has also been successfully used in effectively reducing seizures in pediatric epilepsy [9]. However, increased ketogenesis may have a negative effect on diabetic patients. Diabetic patients are prone to elevated levels of blood ketones because of low basal insulin levels/reduced responsiveness to insulin as insulin is needed to adequately reclear ketones [10,11]. Ketones are organic acids that are ionized at physiological pH; the hydrogen ions thus released bind to bicarbonate causing a drastic drop in serum bicarbonate. As the ketones in the blood increase, and as bicarbonate stores are depleted, metabolic acidosis with a corresponding drop in blood pH ensues, that is, ketoacidosis [5].

Our patient almost entirely excluded carbohydrates from her diet while consuming exogenous ketone esters containing 30 g of D-BHB per serving three times per day. Exogenous D-BHB is directly absorbed into the circulation and quickly increases blood ketone concentrations to 2.8 ± 0.2 mM with just one ketone ester drink, as shown in a study where 15 participants consumed a ketone ester drink which contained up to 24 g of BHB resulting in a decrease of blood pH from 7.41 to 7.31 following one ketone ester drink. In addition, serum bicarbonate was reduced from 23.6 ± 0.7 to 17.0 ± 0.8 mM [4]. Of note, circulating ketone body levels, which in normal individuals are generally <0.5 mM, can reach up to 6-7.5 mM during prolonged fasting and up to 25 mM in cases of severe insulin deficiency [11].

The differential diagnoses including diabetic ketoacidosis, alcoholic ketoacidosis, lactic acidosis, and salicylate toxicity were considered. Given the patient’s normal salicylate and ethanol levels, these etiologies were ruled out. The patient did not have chronic kidney disease, making lactic acidosis secondary to metformin use less likely. Moreover, serum ketones were elevated further, suggesting an alternative cause of acidosis. Therefore, our differential was narrowed down to ketoacidosis caused either by diabetes or consumption of ketones/fasting. Although our patient did have type II diabetes mellitus, her random blood sugar was 176 mg/dL not suggestive of overt diabetic ketoacidosis. In addition, she was not taking any antidiabetic medication of the SGLT2 inhibitor class known to cause euglycemic ketoacidosis. Thus, her ketoacidosis was most likely due to prolonged fasting plus ingestion of exogenous ketone supplements. That notwithstanding, it is plausible that metformin use contributed to her acidosis given that she presented with acute kidney injury and had continued to take her prescribed dose of metformin.

## Conclusions

Here, we presented a case of ketoacidosis induced by keto dieting and consumption of ketone supplements. Although some studies have shown significant weight reduction with the implementation of a ketogenic diet, the adverse consequences of such dieting must be highlighted. As shown in this case, especially in at-risk patient populations such as those with diabetes and/or CKD, the use of exogenous ketone supplements, not often regulated to the same extent as Food and Drug Administration-approved medications, must be cautioned. Our patient was thus counseled on the dangers of taking these weight loss supplements that are unregulated. She was further counseled to follow up with her primary care provider for further education on other safer means to achieve weight loss.
